# A Novel Medium Access Policy Based on Reinforcement Learning in Energy-Harvesting Underwater Sensor Networks

**DOI:** 10.3390/s24175791

**Published:** 2024-09-06

**Authors:** Çiğdem Eriş, Ömer Melih Gül, Pınar Sarısaray Bölük

**Affiliations:** 1 Department of Computer Engineering, Bahcesehir University, Istanbul 34353, Turkey; 2 Informatics Institute, Istanbul Technical University, Istanbul 34467, Turkey; omgul@itu.edu.tr; 3 Department of Artificial Intelligence and Data Engineering, Istanbul University, Istanbul 34134, Turkey; pinar.boluk@istanbul.edu.tr; 4 Department of Software Engineering, Bahcesehir University, Istanbul 34353, Turkey

**Keywords:** underwater acoustic sensor networks, reinforcement learning (RL), energy harvesting, lifetime

## Abstract

Underwater acoustic sensor networks (UASNs) are fundamental assets to enable discovery and utilization of sub-sea environments and have attracted both academia and industry to execute long-term underwater missions. Given the heightened significance of battery dependency in underwater wireless sensor networks, our objective is to maximize the amount of harvested energy underwater by adopting the TDMA time slot scheduling approach to prolong the operational lifetime of the sensors. In this study, we considered the spatial uncertainty of underwater ambient resources to improve the utilization of available energy and examine a stochastic model for piezoelectric energy harvesting. Considering a realistic channel and environment condition, a novel multi-agent reinforcement learning algorithm is proposed. Nodes observe and learn from their choice of transmission slots based on the available energy in the underwater medium and autonomously adapt their communication slots to their energy harvesting conditions instead of relying on the cluster head. In the numerical results, we present the impact of piezoelectric energy harvesting and harvesting awareness on three lifetime metrics. We observe that energy harvesting contributes to 4% improvement in first node dead (FND), 14% improvement in half node dead (HND), and 22% improvement in last node dead (LND). Additionally, the harvesting-aware TDMA-RL method further increases HND by 17% and LND by 38%. Our results show that the proposed method improves in-cluster communication time interval utilization and outperforms traditional time slot allocation methods in terms of throughput and energy harvesting efficiency.

## 1. Introduction

Technological advances in wireless sensor networks and seamless integration with diverse technologies have led to the proliferation of underwater wireless sensor networks and their application areas. Submarine missions are increasingly utilizing underwater sensor gear [1]. Numerous vital applications, such as early warning systems, search and rescue missions, and ecosystem monitoring, can be carried out by these devices. With the collection and analysis of data in aquatic environments, sensors are crucial assets for intelligent systems that enable autonomous decision-making and data analysis. To fully realize the potential of the underwater realm, research focuses on addressing the particular limitations of the underwater medium to enable sustainable data collection in such applications. Underwater sensor nodes primarily employ acoustic communication technology to transmit data over long distances across water. However, acoustic waves have high bit-error rates; therefore, sensors need large transmission powers to avoid packet loss [2]. Considering sensor nodes are typically deployed in sub-sea zones and mostly run on batteries, energy management is crucial for building underwater sensor networks due to maintenance, and human intervention is not practical.

As energy efficiency has been the main focus of underwater communication in the past decade, there have been considerable improvements in this research field to prolong network lifetime by suggesting MAC and routing protocols to improve the utilization of limited energy resources of acoustic sensor nodes to cope with the high energy demand of underwater wireless mediums. The primary reason for energy depletion in UASNs is environmental factors such as acoustic waves. The underwater environment is particularly prone to noise from various sources, such as shipping activities, wind, and aquatic life. Consequently, this leads to a low signal-to-noise ratio (SNR), resulting in a high bit error rate (BER). Since acoustic communication is a dominant technology underwater, the high energy demand of acoustic links is usually decreased by clustering methods. On the other hand, autonomous underwater vehicles (AUVs) are common application assets to gather information collected by clusters in underwater applications.

The use of autonomous robots for data collection is another interesting viewpoint in the literature, and it has been thoroughly investigated in terrestrial wireless sensor networks. By minimizing the energy consumption of cluster heads and UAV robots, several research projects suggested mobile sinks for data gathering from clustered networks [3,4,5,6]. In the study [7], authors explore data collection in a single-hop wireless sensor network, employing a myopic policy based on a Round Robin structure. Findings reveal the suboptimality of this approach for various energy harvesting processes, emphasizing limitations while achieving optimality in specific instances. Yet, in comparison to terrestrial wireless sensors, the underwater system has higher financial costs, even though the assistance of autonomous robots lowers the energy cost of sensor equipment.

To maximize UASN lifetime, several studies in the literature focus on improving network clustering and cluster head selection methods [8,9,10]. Furthermore, recent studies present that harvesting ambient energy sources is a promising method to improve the life cycle of underwater sensors [11,12,13,14]. Recent studies [15,16] have demonstrated that UASN applications must optimize energy management in routing by using ambient energy to provide sustainable data collection from underwater sensor nodes. The main obstacle to optimizing energy management in submerged communication channels is the constraints of sensing apparatus and harvesting machinery.

In our previous study [17], we proposed a cluster head selection method for energy-aware routing problems in UASNs and investigated how expected available energy affects cluster head selection while accounting for the stochastic energy harvesting techniques of individual sensors. We tackle the same problem and propose a novel reinforcement learning-based algorithm for determining cluster heads (CHs), which involves sensor nodes’ positions and remaining energy in addition to estimated harvested energy [18]. Numerical results validate that our introduced approach significantly increases harvested energy and, hence, extends the network’s operational lifetime considerably, proving that energy harvesting awareness improves the operational lifetime of underwater sensors.

In this study, we introduce a time slot scheduling policy and improve the medium access control (MAC) protocol designed specifically for energy harvesting underwater acoustic sensor networks (EH-UASNs). The main goal of this policy is to optimize the network’s lifespan while enhancing throughput. We utilize a clustered topology, leveraging its duty cycling and data aggregation nature, to enhance the efficiency of network energy utilization. We investigate the time division multiple access (TDMA) scheme for intra-cluster communication. TDMA optimizes energy usage by enabling inactive nodes to transition into sleep or idle modes until their allocated time slot for communication arrives. Our main goal is to enhance the efficiency of energy harvesting within clusters of the network by leveraging the allocation of time slots. We introduce a novel cooperative multi-agent reinforcement learning algorithm to schedule time slots of the TDMA MAC protocol for EH-UASNs to maximize harvested energy and enhance throughput through adaptive time slot allocation in intra-cluster nodes.

Our main contributions can be listed as follows:

- We propose a novel method for TDMA time slot allocation where nodes independently learn from their ambient energy resources and harvesting opportunities in order to autonomously decide their communication slots in their cluster. In our method, we model the TDMA time slot allocation task as a multi-armed bandit problem on individual nodes. We combine multi-armed bandit problem with a cooperative and independent Q-learning algorithm to maximize harvested energy in the network and mitigate the continuous scheduling duty on the cluster heads.
- To analyze the efficiency of our proposed method in realistic channel conditions, we optimized the power consumption of the sensor node to maintain connectivity in clusters via optimal carrier frequency selection according to the channel parameters and distance to the receiver.

The remainder of this paper is organized as follows: Section 2 presents the related work. Section 3 gives the system model; then Section 4 presents the problem statement. Section 5 proposes our novel approach. Section 6 evaluates the performance of the proposed algorithms numerically. Section 7 further investigates the performance of our approach. Section 8 concludes our work; Section 9 gives future work.

## 2. Related Work

There is  growing literature about medium-access strategies to improve energy efficiency in underwater wireless sensor networks. In wireless sensor networks, the aim of MAC protocols is to decrease latency in the network and focus on the management of high traffic, which is extensively studied [19]. These efforts can be categorized as contention-based and contention-free protocols. In contention-based protocols, all nodes can transmit data packets simultaneously without any predefined scheduling algorithm. In [20], a contention-based MAC protocol, TDTSPC-MAC, is developed for three-dimensional UASNs facing challenges such as long propagation time, high bit error rate, and limited bandwidth. The protocol integrates time synchronization, power control, clustering, layering, and sleep mechanisms. TDTSPC-MAC utilizes hierarchical division of three-dimensional space and a distributed clustering algorithm, combining time synchronization and power control strategies to prevent collisions and reduce energy consumption through monitoring and sleep mode. Yet, in scenarios involving extensive data transmission or a significant number of nodes, data packets become susceptible to collisions. Large transmission delays and limited transmission rates inherent in underwater sensor networks increase collision probability and cause low energy utilization. In [21], a delayed-reward ALOHA-Q protocol is designed for UASNs. By leveraging Q-learning, DR-ALOHA-Q autonomously determines optimal time slots and offsets for data transmissions to improve channel utilization in the presence of large propagation delays. Contention-based methods require active listening and short control packet exchanges in order to mitigate long idle waiting times and improve overall energy consumption and transmission delay in UASNs. In [22], challenges in underwater sensor networks related to high latency, low bandwidth, and high bit error rate in acoustic communication are investigated. It highlights the limitations of contention-based medium access control (MAC) protocols in UASNs and proposes a novel contention-free MAC protocol called DL-MAC. The DL-MAC protocol addresses underwater MAC challenges through depth-based layering, a distributed clustering algorithm, and TDMA-based scheduling, demonstrating superior performance in terms of throughput, packet delivery ratio, energy consumption, and packet loss.

On the other hand, contention-free MAC protocols can easily address the well-known hidden node problem without extra control packet overhead and interference of neighboring nodes by utilizing scheduling nodes to distinct time slots to perform transmissions. A well-known contention-free MAC protocol, TDMA, attracted researchers, while other contention-free protocols, such as FDMA, suffer from limited available bandwidth, and CDMA faces challenges due to the near-far problem [23]. Hence, TDMA-based MAC protocols have been the main focus for long-term surveillance applications in the literature. Numerous TDMA-based medium access methods are proposed [24,25,26] to leverage the benefits of simplicity, fairness, and energy efficiency by mitigating issues such as collisions, idle listening, and transmission over-hearing. However, TDMA-based MAC protocols exhibit certain drawbacks, such as suboptimal channel utilization, challenges in dynamic scalability, and the need for time synchronization. Wang et al. [27] proposed an online scheduling algorithm to calculate the optimal action, namely, the decision of transmitting the packet or holding it back in the packet queue in each time slot, with the aim of decreasing energy consumption in packet transmission. In this work, a channel state prediction algorithm is employed in order to determine the optimal action. Aiming to minimize energy consumption and considering the channel conditions in transmissions, a transmission strategy is proposed in [28]. Ref. [29] suggested channel-aware transmission scheduling, considering the duration of each slot and channel conditions and adjusting the amount of data to be transmitted.

In [30], a deep reinforcement learning (DRL) algorithm is introduced, delayed-reward deep Q-network (DR-DQN), to improve throughput degradation due to long propagation delays in underwater acoustic networks (UANs). Delayed-reward DRL multiple access (DR-DLMA) is proposed, aiming for an optimal channel access strategy that maximizes network throughput by efficiently utilizing available time slots resulting from long propagation delays. In [31], authors suggest prediction of the channel state by capturing temporal and frequency correlations in a one-dimensional convolutional neural network (CNN). In [32] a time slot allocation scheme for clustered underwater acoustic sensor networks, utilizing physical layer information to minimize energy consumption caused by unnecessary retransmissions. This enhancement aims to extend network lifetime and improve throughput. To streamline the process and reduce overhead and computational complexity. In their two-phase approach, first each member node autonomously determines the number of required time slots for the next intra-cluster cycle by solving a Markov decision process (MDP). Second, the cluster head optimizes scheduling decisions based on channel quality and an urgency factor.

Despite ongoing research efforts, existing studies mainly focus on the improvement of throughput and energy utilization, considering only the channel state and fairness. On the other hand, the impact of energy harvesting in underwater medium has proven its importance in the design of communication protocols by extending the operational lifetime of underwater sensor devices considerably [14]. A noteworthy study conducted by Ge, Y. et al. [16], focused on the improvement of throughput and energy harvesting for solar-powered and clustered WSNs. This work proposes a multi-agent reinforcement learning algorithm to maintain nodes in an energy-neutral state by balancing the consumed and harvested energy of nodes. Yet, this study focuses on the WSNs, their peculiar characteristics underwater, and the amount of available ambient energy that needs to be taken into account for UASNs. A decentralized reinforcement learning approach for random access is proposed to improve the throughput of the network, considering energy harvesting for sustainable data collection from EH-UASNs [15]. In this work, a multi-agent cooperative learning algorithm is used in the adaptive choice of contention window size to optimize throughput while maintaining fairness in the network. However, the authors focused on the throughput and the reward function, considering only the fairness and throughput of the network. Increasing energy harvesting utilization is not investigated in this study.

When considering clustered UASNs and TDMA-based protocols, it is imperative to allocate resources effectively to ensure reliable and efficient operation. Therefore, time slot allocation methods for underwater sensor networks need to be robust and accurate, as this greatly affects communication efficiency. Extensive research has been conducted on medium-access strategies aimed at enhancing energy efficiency in UASNs. Notable focus has been placed on reducing network latency, managing traffic, and minimizing energy consumption through various mechanisms such as time synchronization, power control, and sleep modes. However, these studies focus on optimizing energy efficiency rather than maximizing energy harvested from the environment. In the literature, a significant gap persists regarding MAC protocols specifically tailored to optimize and maximize energy harvesting in UASNs, accounting for the distinctive challenges posed by underwater environments. To the best of our knowledge, existing literature on MAC protocols in UASNs has mainly concentrated on enhancing network efficiency and optimizing energy usage; no prior study was specifically aimed at optimizing energy harvesting through channel access strategies. Our method combines multi-armed bandits with cooperative, independent Q-Learning and proposes a decentralized and robust solution that adapts to dynamic environments with minimal communication overhead.

## 3. System Model

Our system comprises 100 acoustic sensor nodes anchored to the multi-level depths across the seabed and designed to be used for long-term underwater monitoring applications. Sensor measurements such as pressure, water salinity, and temperature are relayed to a gateway (sonobuoy) on the sea surface. Nodes are able to maintain a connection to the gateway via one-hop. However, due to excessive energy depletion in data transmission over long distances, we considered a clustered network and performed a data collection task via elected cluster heads. We assume interference-free communication in inter-clusters with the use of different carriers or orthogonal spreading codes. All nodes in the network have energy harvesting equipment, and nodes perform energy harvesting only in their idle times, assuming independently and identically distributed (i.i.d.) harvesting states.

### 3.1. Network Model

**Figure 1:**
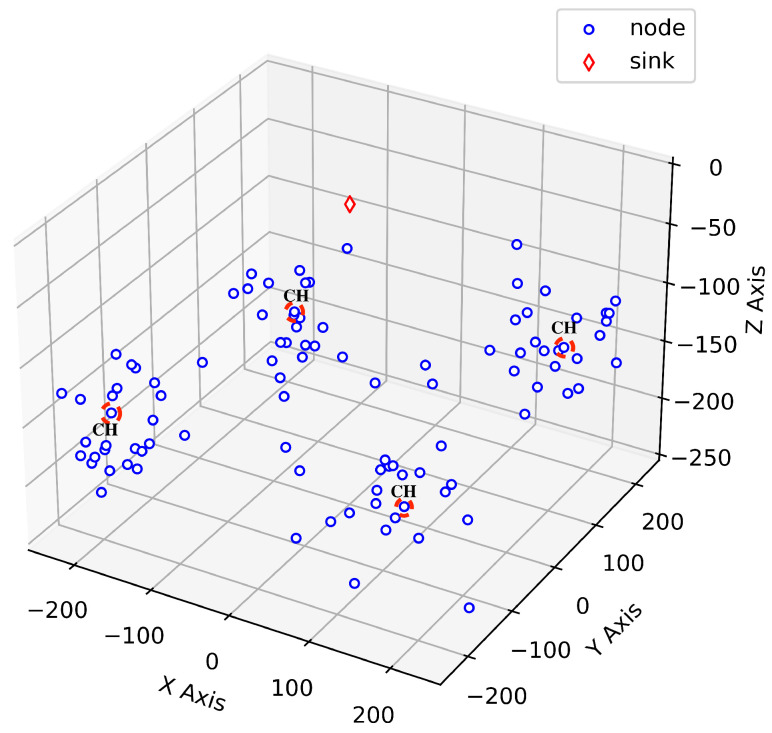
3D Sample network topology showing gateway and sub-sea node placements.

We model the three-dimensional non-uniform distribution of 100 acoustic sensors ($N_{node}=100$) randomly deployed in 250 m × 250 m × 250 m. There is one surface sink on the sea surface, at the top of the network. Nodes are fixed to the ocean floor, and we presume that sensor locations will not change over time. A sample 4-clustered 3D network topology consisting of 100 underwater sensor nodes is shown in Figure 1. Cluster members are connected to their cluster heads via single hop.

Data aggregation and the transfer of aggregated data to the gateway are handled by cluster heads. There are several rounds to complete this operation. A frame consists of one packet of *L* bits transmitted by each node, and a round is divided into many successive frames. Keep in mind that nodes use energy for data transmission, reception, and aggregation. The length of time that a packet is transmitted and received is shown by $\tau_{tx}$ = L/R, where *R* is the sensor nodes’ bit rate. Moreover, all nodes harvest energy only in their idle periods.

In our system, the network is divided into 4 regions by the k-means clustering method. The surface gateway divides the network into k clusters by computing the elbow method and initializing clusters [18]. Each node in the network learns their clusters from the broadcast message of the gateway station. As the next step, each node determines whether to become the cluster head for the current round. With prior knowledge of members in its cluster, cluster heads assign a unique time slot for each node in the cluster. Cluster members send their data to the cluster head in their programmed time slots to avoid collisions. After data transmission, each node becomes idle and harvests ambient energy until the next frame of data collection.

Given that underwater channels have limited capacity and high propagation delay, TDMA-based medium access protocols are widely used by cluster-based data collection [33]. These protocols have been chosen over collision avoidance-based protocols due to their poor performance in underwater acoustic networks [34]. Furthermore, time slot allocation has the potential to reduce energy usage by preventing contention and collisions among packets without extra overhead for channel reservation that requires short control packets.

### 3.2. Physical Layer Model

Attenuation and absorption have a significant impact on acoustic waves over long distances. To determine transmission power in acoustic modems, an underwater propagation model is presented. Depending on the location of the destination node, the source node modifies its power. Sonar equations and the propagation of underwater acoustic waves are defined in [35], and the acoustic intensity of the transmitted signal is determined as [36].

(1) $$SL{\left(d,f\right)}_{ij}=A{\left(d,f\right)}_{ij}+N\left(f\right)+\gamma {\left(d\right)}_{dB}+DI$$

where $SL{\left(d,f\right)}_{ij}$ represents the sound intensity magnitude emitted by node-i at a distance *d* (in meters) in relation to the acoustic pressure of sound (dB ref μPa) and is calculated using the acoustic signal frequency *f* (in kHz). Path loss of acoustic waves at frequency *f* is represented by $A{\left(d,f\right)}_{ij}$, which increases with node distance *d* and is quantified as dB in [37]

(2) $$A{\left(d,f\right)}_{ij}=10\kappa \log_{10}\left(d_{ij}\right)+\alpha \left(f\right)\times d_{ij}\times 10^{-3}$$

where κ denotes the spreading factor; the absorption coefficient is expressed as α(f) given as dB/km in Equation (3) [38]

(3) $$\alpha \left(f\right)=\frac{0.11f^{2}}{1+f^{2}}+\frac{44f^{2}}{4100}+f^{2}+2.75\times 10^{-4}f^{2}+0.003$$

The ambient noise level is computed in dB ref μPa accounting for the cumulative noise levels induced by water turbulence (i.e., $N_{t}\left(f\right)$), surface ships (i.e., $N_{s}\left(f\right)$), thermal activities (i.e., $N_{th}\left(f\right)$), and breaking waves (i.e., $N_{w}\left(f\right)$) defined in [37].

(4) $$N\left(f\right)=N_{t}\left(f\right)+N_{s}\left(f\right)+N_{th}\left(f\right)+N_{w}\left(f\right)$$

The bit error rate (BER) is defined for 16-QAM modulation with orthogonal frequency division multiplexing (OFDM) encoding in Equation (5). The signal-to-noise ratio (SNR) can be calculated as follows in order to maintain a BER on a link (i.e., $10^{-9}$) [34]

(5) $$p_{ij}^{b}=\frac{3}{8}erfc\left(\sqrt{\frac{4}{10}\frac{B_{N}}{R}\gamma {\left(d\right)}_{ij}}\right)$$

where $B_{N}$ is noise bandwidth (in Hz), $\gamma {\left(d\right)}_{ij}$ is SNR in linear form, and *R* is data rate in bps. The intensity level of sound ($I_{ij}^{tx}$) at a distance *d* is used to calculate the electrical power needed for transmission [34]

(6) $$p_{ij}^{tx}=I_{ij}^{tx}\times 2\pi \times 1m\times h_{i}$$

where $h_{i}$ represents the sea depth of node-i in meters. The source level ($SL{\left(d,f\right)}_{ij}$) is used to calculate the intensity of the acoustic signal ($I_{ij}^{tx}$) [34].

(7) $$I_{ij}^{tx}=10^{\frac{SL{\left(d,f\right)}_{ij}}{10}}\times I_{0}$$

$I_{0}$ is the reference intensity at 1 m defined as $0.67\times 10^{-18}$ (Watts/m^2^) [34]. To satisfy the predetermined BER and SNR threshold, the required transmission power is calculated with respect to the distance between the source and destination. Given electrical transmission power ($p_{ij}^{tx}$), the total energy consumption of the node transmitting a single data packet is calculated

(8) $$E_{ij}^{t}=p_{ij}^{tx}\times \tau_{tx}$$

where $\tau_{tx}$ is duration of single packet transmission determined as $\tau_{tx}=L/R$.

### 3.3. Stochastic Model of Piezoelectric Energy Harvesting

Piezoelectric harvesters are triggered by the change of energy in flowing water. This harvester makes use of the flow’s turbulence, which forms vortexes with varying rotational velocities and opposing directions. The underwater environment is characterized by high levels of variability and uncertainty. Sensor nodes are distributed across various depths, encountering a range of environmental factors that cause variations in the produced electrical energy. The pressure gap in clockwise and counter-clockwise directions is calculated using the density (ρ) and velocity of the flow as ($v_{f}$) [11]

(9) $$\varrho_{cw}=\frac{\rho }{2}v_{f}^{2}$$

(10) $$\varrho_{ccw}=-\frac{3}{2}\rho v_{f}^{2}$$

The amount of electrical power produced by a piezoelectric harvester varies depending on the pressure gap, piezo-material, and cantilever properties. The piezoelectric harvester’s generated electrical energy ($W_{el\_cw}$ and $W_{el\_ccw}$) in both clockwise and counter-clockwise directions is calculated [11]

(11) $$W_{el}=\frac{1}{128}\varrho^{2}\frac{{d_{31}^{p}}^{2}}{\varepsilon_{0}\varepsilon_{r}}\frac{BL^{5}}{T_{pzt}^{3}}$$

where ${d_{31}^{p}}^{2}$, $\varepsilon_{0}$, and $\varepsilon_{r}$ indicate the piezoelectric constant, absolute permittivity, and relative permittivity of the piezo-material, respectively. *B*, L=2.1D, and $T_{pzt}$ indicate the cantilever width, length, and thickness respectively.

As the flow velocity changes, we use the Poisson distribution to model the turbulence of the flow patterns. The mean velocity of the flow is observed in multi-level depths of the Bosphorus strait [39]. The vortex frequency at a time *t*, $f_{v}$, varies with respect to the velocity of the flow ($v_{f}$), bluff size (*D*), and Strouhal Number ($S_{t}$) is determined as [11]

(12) $$f_{v}\left(t\right)=\frac{S_{t}\times v_{f}\left(t\right)}{D}$$

Given Poisson events, the harvested energy of a node *i* is calculated for *t* = 0, …, *T*

(13) $$E_{i}^{h}\left(T\right)=\int_{t=1}^{T}2f_{v}\left(t\right)\left(W_{el\_cw}\left(t\right)+W_{el\_ccw}\left(t\right)\right)dt$$

## 4. Problem Definition

As with TDMA-based and round-robin methods in clustered sensor networks, each transmission round is divided into frames, f. In each frame, cluster members send a fixed-length (L) data packet to their cluster head. We consider a data backlogging system where nodes capture measurements for long-term monitoring applications and always have data to transmit when it is their turn. In long-term monitoring applications, we consider a clustered network, assuming bi-directional communication in cluster heads to operate control signals and data aggregation. Additionally, to evaluate energy efficiency in these applications, low latency and high reliability are considered secondary concerns. This approach allows for the sustainability of the network over long periods, prioritizing energy efficiency over quality of service (QoS) and ensuring data integrity at the cluster head level. All nodes have the same data rate and are allowed to send the same number of packets in each frame. We use transmission power control (TPC) so that each transmission is held with a transmission power of nodes to satisfy a predetermined SNR for successful packet reception [34].

We consider a network of non-uniformly dispersed 100 nodes in varied depths underwater. Nodes maintain channel access in TDMA fashion, communicating with cluster heads over their scheduled time slots per frame. Cluster heads determine the time intervals and notify cluster members of their time slots for data transmission.

We denote the set of *N* acoustic sensor nodes by $S=\{s_{1},s_{2},\ldots ,s_{N}\}$. We assume stochastic energy harvesting processes modeled by Poisson distribution at each node.

The remaining energy and the amount of harvested energy of a sensor node $s_{i}$ in a time slot *t* denoted as $E_{i}\left(t\right)$ and $E_{i}^{h}\left(t\right)$, respectively. $E_{i}^{h}\left(TS\right)$ represents total harvested energy per frame. Considering frames consist of TS many transmission slots, the same as the number of nodes in clusters, the transmission slot set is represented as $\mathrm{TS}=\{t_{1},t_{2},\ldots ,t_{N}\}$, the total amount of harvested energy in cluster within time steps from 1 to TS in frame, under a policy π where $t_{tx_{i}}$ is the designated time slot index of sensor i, $1_{\left\{t_{tx_{i}}\right\}}$∈{0,1} is the indicator function, and harvested energy at time slot $t_{m}$ can be written as $E_{i}^{h}\left(t_{m}\right)=E_{i\left(t_{m-1}\right)}^{h}1_{\left\{t_{tx_{i}}\right\}}$. Overall harvested energy under a policy is determined as

(14) $$V_{i}^{\pi }\left(TS\right)=\sum_{i=1}^{N}V_{i}^{\pi }\left(TS\right)=\sum_{i=1}^{N}\sum_{t=1}^{TS}E_{i}^{h}\left(t\right)$$

where $V_{i}^{\pi }\left(TS\right)$ is the sum of harvested energy under policy π in the cluster from starting time slot 1 through *TS*

(15) $$E_{i}^{h}\left(TS\right)=\sum_{t=1}^{TS}\left[E_{i}^{h}\left(t\right)-\sum_{t=t_{tx_{i}}}^{t_{tx_{i}}+1}E_{i}^{h}\left(t\right)1_{\left\{t_{tx_{i}}\in TS\right\}}\right]$$

Our objective is to maximize the total amount of harvested energy by each node over a finite time horizon,

(16) $$\max_{{\left\{\pi \left(t\right)\right\}}_{t=1}^{T}}\frac{1}{T}E\left[V_{i}^{\pi }\left(T\right)\right]$$

In clustered networks, optimal time slot scheduling algorithms employ cluster heads to schedule the transmission slots of each node. However, the cluster head may not select the optimal transmission slot without incurring communication overhead to gather knowledge on the energy harvesting states of nodes. Even when this information is provided to the cluster head, its timeliness might be compromised due to costs associated with complexity and computational delays due to slow convergence. Adopting a global optimal solution approach is not feasible. Therefore, instead of relying on centralized methods, delegating the transmission slot allocation task to cluster members not only delegates autonomy but also unveils the harnessing of ambient energy through adaptive transitions between transmission and idle energy harvesting modes. On the other hand, distributed reinforcement learning and game-theoretic algorithms offer independent learning with increased communication overhead and computational complexity. However, these methods compromise scalability at the expense of additional communication overhead and lack adaptability in dynamic environments. We model this problem as multi-armed bandits and combine the cooperative, independent Q-learning approach by leveraging shared rewards with environmental signals. In our model, slot selection with cooperation is inspired by Independent Q-Learning, aiming to balance individual optimization with cooperative behavior. Our proposed reward structure guides agents towards cooperation while maintaining independent learning at each node. Nodes can determine their slots to transmit packets, incorporating environmental conditions to determine the optimal timing for packet transmission or reverting to energy harvesting mode. In our model, we used the stochastic piezoelectric harvester, and the following assumptions were made:

- The statistical characteristic of all random quantities (flow velocity, voltage peaks, etc.) is known by observation and experiment [11,39].
- Harvested power in time slots is independent and identically distributed.
- As a well-known practice, we employ two different MAC methods on the low density (size of inter-cluster nodes) and high density (size of intra-cluster nodes) settings in clustered network topology. Namely, the TDMA MAC protocol for intra-cluster communication and the CSMA MAC protocol for determining the average power consumption of cluster heads in inter-cluster communication.

## 5. Transmission Slot Selection Policy with Reinforcement Learning

The underwater medium introduces uncertainties regarding water depth and environmental conditions, impacting the magnitude of underwater vibrations and movements and thereby influencing the harvested energy over time. Scheduling methods based on time slot allocation often overlook environmental factors that influence the availability of harvestable energy in the underwater environment when assigning time slots to nodes within the cluster. For this reason, nodes transmit within their scheduled time periods, regardless of available energy, resulting in a deficiency of the expected amount of harvested energy.

In our model, we adapt a multi-agent independent Q-learning algorithm with cooperation running multi-armed bandit problem on each node to autonomously select their time slot per data frame and learn from their past decision-making choices influenced by ambient conditions. In the single-agent, multi-armed bandit problem, the agent makes a choice among *k* different actions modeled as arms of a slot machine [40]. In this slot machine, each time after an action is performed, the agent receives a reward depending on a probability distribution in arms. The value of the actions is recorded as the mean of rewards over time steps. The agent maintains the estimated value of each action *a* in each time step, as $Q_{t}\left(a\right)$. The aim of the agent is to maximize the cumulative rewards over repeated actions in time steps.

In a single data frame, $R_{t}\left[a_{i}\right]$ denotes the reward received after action $a_{i}$ is selected in time step *t*, and the estimated value of action $a_{i}$ after t−1 is $Q_{t}\left[a_{i}\right]$ defined in Equation (17).

(17) $$Q_{t}\left[a_{i}\right]=Q_{t-1}\left[a_{i}\right]+\frac{1}{t-1}\left(R_{t-1}\left[a_{i}\right]-Q_{t-1}\left[a_{i}\right]\right)$$

**Figure 2:**
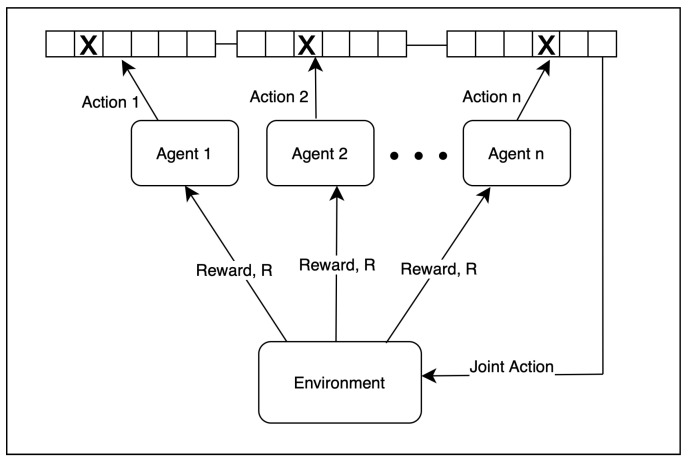
Training paradigm of multi-agent reinforcement learning system.

On the other hand, multi-agent systems (MASs) focus on developing algorithms that enable agents to learn adaptively and optimize their behavior in changing environments. Multi-agent reinforcement learning (MARL) aims to educate multiple agents simultaneously within a common environment by employing reinforcement learning (RL) techniques. In a fully cooperative setting, agents work together toward a shared goal, like assembling machines [41]. Figure 2 represents the multi-agent system, *n* distributed agents as underwater sensor nodes, cooperatively play the same multi-armed bandit game. Each agent has its own record of the Q table as time slot arrays. Agents choose actions independently, constructing a joint action array from received packets at the cluster head. We model k arms to represent time slots, the same as cluster size (the total number of cluster members). Note that each frame consists of k, i.e., TS many time slots. To find the best global combination of arms, agents wish to maximize a global return, or, in other words, minimize the overall regret. Associated with arms, mutually independent sequences of i.i.d.-valued rewards are modeled. Specifically, the reward is the overall energy gained in the rest of the arms except the arm selected for transmission. We run our model for a predetermined duration of a time slot. A detailed explanation of our method is given in Algorithm 1.
**Algorithm 1** Distributed Multi-Agent Learning Algorithm for Transmission slot selection**Require:** 0⩽γ⩽1, α∈(0,1], small ε>0, n≥0   Initialize $Q_{i}\left[a\right]\leftarrow 0\forall a\;\in A_{i}$ and ∀i∈N   Update Q table $Q_{i}\left[a\right]\leftarrow E_{i}^{h}\left(t\right)\forall t\in \mathrm{TS}$   **for** each time slot **t** in frame (TS) **do**         **for** each node **n** in ${\mathrm{Cluster}}^{k}$ **do**           **   if** random(n)≥ε **then**                   Choose exploitation                   Select maximizing action $a_{i}=\arg\max Q_{i}\left[:\right]$           **   else**                   Choose exploration                   Select a random action $a_{i}$              **end if**              node **n** selects action $a_{i}$ forms the joint action **a**$\leftarrow \langle a^{1},\ldots ,a^{N}\rangle$               Receive local reward from environment $R_{t}^{n}\left(a_{i}\right)$         **end for**         Return global reward from environment $R_{t}^{N}\left(a\right)$         $R_{t}\left(a_{i}\right)\leftarrow \beta R_{t}^{N}\left(a\right)+\left(1-\beta \right)R_{t}^{n}\left(a_{i}\right)$         Update $Q_{t}\left[a_{i}\right]\leftarrow \alpha *\left(R_{t}\left(a_{i}\right)+\gamma *Q_{t}\left[a_{max}\right]-Q_{t-1}\left[a_{i}\right]\right)$   **end for**

1. Agent: Every underwater sensor node in the network is an agent.
2. Action: The action is the selection of a transmission slot to maximize the harvested energy during the current frame of data transmission. A transmission slot can be selected depending on the interaction of each sensor with its environment, which affects the available energy. Transmission slots are designed as discrete time intervals. Action is the choice of the time interval for data transmission.
3. Reward: The reward calculation contains the weighted sum of two parts, namely the mutual reward and the independent local reward. The mutual reward of a cluster member after selecting a transmission slot is the total number of data packets that arrive in the cluster head $R_{CH}$, and it is broadcast to cluster members at the end of each frame. The weight coefficient β is fixed at 0.4. Reward function penalizes conflicted slot selection with the decreased amount of received packets. In case of conflict, nodes will wait for the next round to send their data packets. As a result, a reduction in the number of received data packets will lead to a decrease in the global reward, prompting nodes to adjust to their neighboring nodes’ decisions. The total harvested energy of the cluster member serves as the agent’s local reward, calculated as the overall energy gain excluding the selected time interval for transmitting a single data frame. In simpler terms, it represents the accumulated harvested energy within the cluster during the idle time of nodes in the previous frame.
  (18) $$Reward_{n}=\beta \sum_{t=1}^{TS}R_{CH}+\left(1-\beta \right)\sum_{t=1}^{TS}E_{s_{n}}^{h}\left(t\right)$$

We propose a multi-agent reinforcement learning algorithm for the optimal policy in the selection of time slots. Awareness of environmental conditions and observed energy fluctuations in ambient resources can be utilized in the determination of timing for data transmission; hence, this approach potentially alleviates the load on the cluster head. Furthermore, the selection of the best transmission slots for each node in the cluster is of mutual interest to all nodes in the system, thereby maximizing energy utilization in the cluster. With learning, each sensor can exploit the ambient resources to harvest energy to assure the maximal lifetime of the network. In our method, we consider round-based data collection. After one frame of data collection is complete, all nodes in the cluster take the same reward for the number of data packets received and a local reward for the sum of their harvested energy in the frame.

For intra-cluster communication, nodes perform data transmission in their time slots that are designated by their cluster head and switch back to an idle state until the next frame. However, instead of centralized control of medium access, nodes determine their slots by learning the environmental conditions of harvesting parameters.

**Figure 3:**
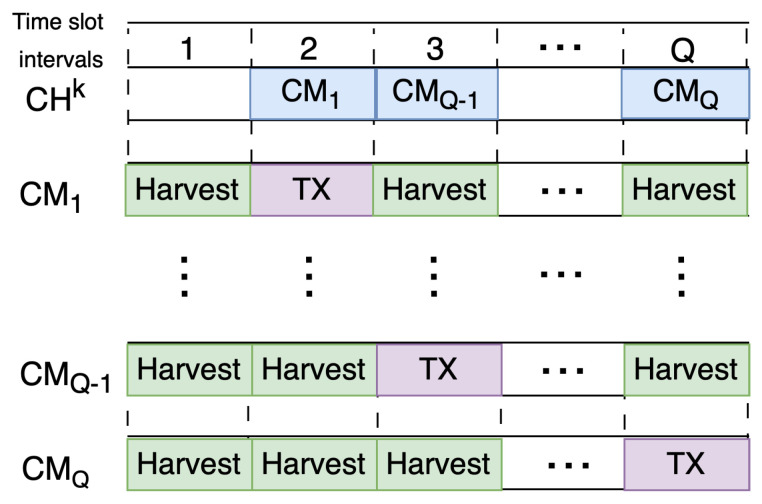
Example self scheduling of transmission slots representing high/low harvesting conditions.

In Figure 3, there is an example timeline showing our approach within a data frame in clustered networks. A round’s duration is preset to guarantee that, on average, every node can function as both a cluster head and a non-cluster head several times over its lifetime [9]. $CH^{k}$ represents the cluster head of *k*th cluster with a time slot allocation table and contains record of each packet reception from its cluster members. Here, cluster members, denoted as CM, decide their transmission slot for a packet by selecting one of *Q* available time slots in current frame. Nodes observe energy harvesting rates and schedule their transmission to a time slot with less available energy compared to other slots. For instance, $CM_{1}$ decides allocating time slot 2 according to its available energy. Depending on its value of Q table for time slot 2, the transmission decision is calculated based on its harvested energy in previous frame, with the lowest in time slot 2.

## 6. Numerical Results

In this section, we compare the efficiency of our transmission slot scheduling method with the traditional TDMA approach. Additionally, the contribution of energy harvesting to the lifetime of a clustered underwater sensor network is also denoted in our Python simulations. We used the piezoelectric energy harvesting model [11] with the underwater communication channel model [34] and round-based data collection in clustered networks is adopted from [42]. We assume data is backlogged, i.e., nodes always have data to transmit to their cluster heads. Data packets are fixed in size, and transmission power is adjusted according to the channel conditions and distance of cluster members to their respective cluster heads. We present a performance comparison of 100 different network topology settings in this section. Simulation parameters are defined in Table 1.

In TDMA-based medium access methods, the transmission slot of each node in the cluster is organized by the cluster head, and cluster members are notified about their scheduled slots for communication. We investigate the performance of TDMA and the effect of piezoelectric energy harvesting on the clustered underwater sensor network. The TDMA-none curve indicates 100 nodes without energy harvesting equipment and using TDMA slot allocation for in-cluster communication. In TDMA-none, the curve cluster head schedules the transmission slots for cluster members. The TDMA-EH curve presents 100 nodes with piezoelectric harvesting equipment and the same transmission slot scheduling method as TDMA-none. We present the performance of our method in the TDMA-RL curve, the transmission slot scheduling with cooperative learning, and compare its efficiency to the traditional TDMA method.

**Figure 4:**
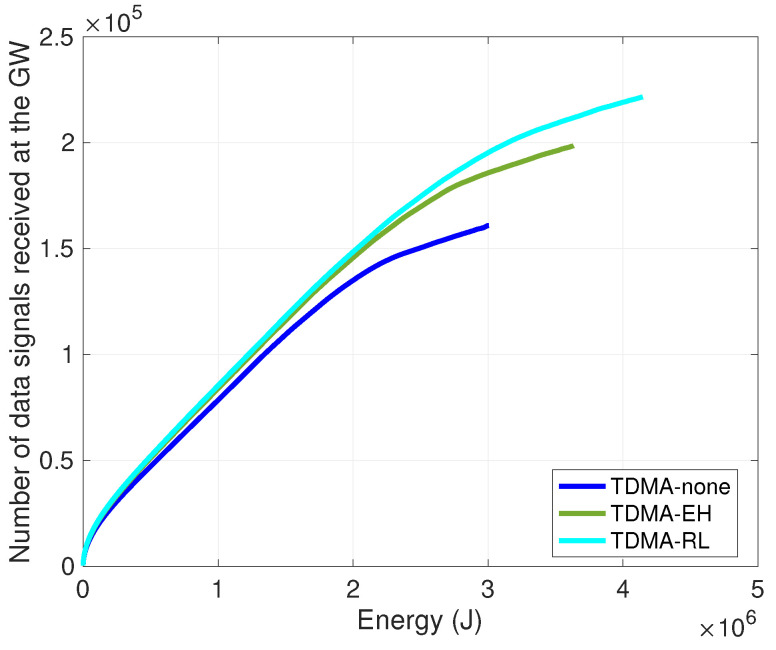
Number of data signals received at the gateway per energy.

Figure 4 depicts that our proposed method improves energy utilization as more data signals are received by the gateway station. In the TDMA-none curve, nodes perform transmissions in predetermined slots scheduled by cluster heads without energy harvesting equipment. The TDMA-EH curve incorporates energy harvesting. It is observed that nodes collect more energy as in consecutive rounds. Therefore, compared to the TDMA-none curve, the TDMA-EH curve sends 20% more data signals to the gateway. The TDMA-RL curve denotes the performance of our approach. As available energy changes during idle-tx states in frames, each node experiences varying levels of energy within its surrounding environment. As the selection of transmission slots adapts to the harvesting conditions, the potential for collecting more energy during each frame increases. It is observed that 15% more data signals can be received by gateway in the TDMA-RL curve compared to the TDMA-EH curve. However, in the traditional TDMA slot scheduling, nodes are unaware of their ambient conditions, and energy is harvested in pre-established slots during frames; compared to the TDMA-none curve, it is observed that ∼35% more data packets can be transmitted by exploiting the available energy underwater.

**Figure 5:**
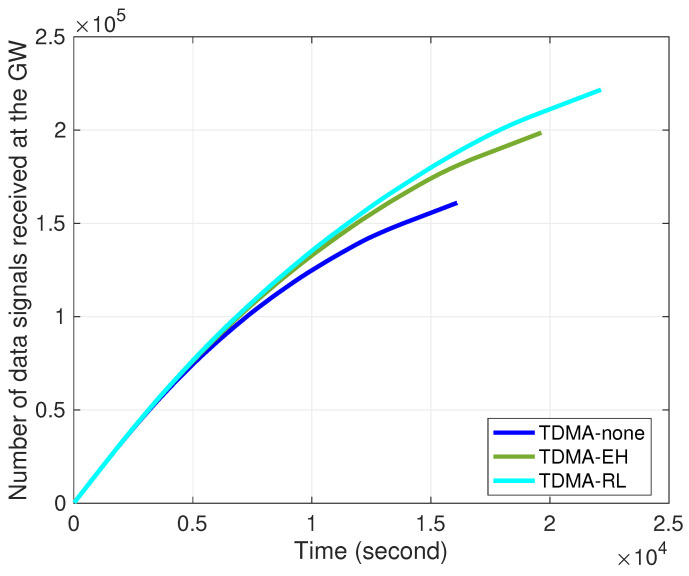
Number of data signals received at the gateway over time.

Figure 5 represents the performance of the previously mentioned methods in terms of throughput. It is observed that at the late stages of simulation in the TDMA-RL method, more data signals are received per unit of time compared to the TDMA-none and TDMA-EH methods due to better utilization of available energy. It is also observed that all curves are identical in the early stages of the simulation. In the forthcoming rounds, more energy is collected while nodes await idle and harvest energy until their communication slots in the cluster. Compared to the TDMA-none method, in TDMA-EH and TDMA-RL, as more nodes remain active in rounds, 21% and 37% more data packets can be relayed to the gateway, respectively. The TDMA-RL and TDMA-EH curves have the same characteristics, but they collect more energy in successive rounds. On the other hand, the TDMA-RL curve outperforms by selecting efficient slots to remain idle, observing the ambient available energy, selecting the most inefficient time slot in terms of available energy, and performing data transmission. Consequently, TDMA-RL captures more energy, whereas the TDMA-EH method neglects to exploit available energy by transmitting data during periods of high energy availability in time slots.

**Figure 6:**
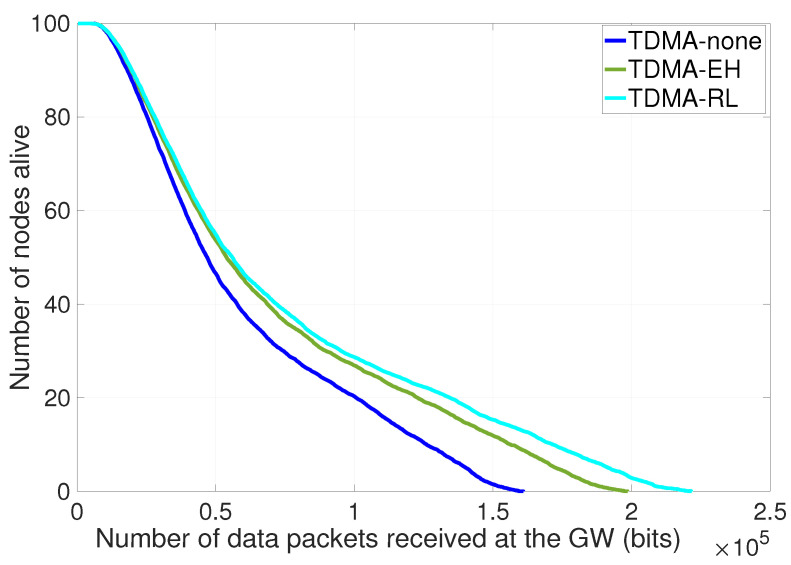
Number of alive nodes per data items received.

Figure 6 shows the number of available nodes over data signals received at the base station. As more energy is captured from environment, lifetime duration of nodes is improved and more data can be generated compared to TDMA-EH utilizing traditional TDMA approach. In TDMA slots, nodes communicate with their cluster heads without being aware of their ambient energy conditions. Therefore, less ambient energy can be collected. In the early phase of the simulation, it is observed that three methods have the same decrease rate in the number of alive nodes until 5% of nodes run out of energy and a few megabytes of data are received at the base station. It is observed that if energy harvesting (EH) is involved in the system, the amount of data packet reception increases by 23% regardless of the medium access method. It is clear that harvested energy significantly contributes to the battery of nodes in successive rounds; hence, nodes can perform more data transmission. The TDMA-RL curve improves the amount of data packets by 11% compared to the TDMA-EH method.

**Figure 7:**
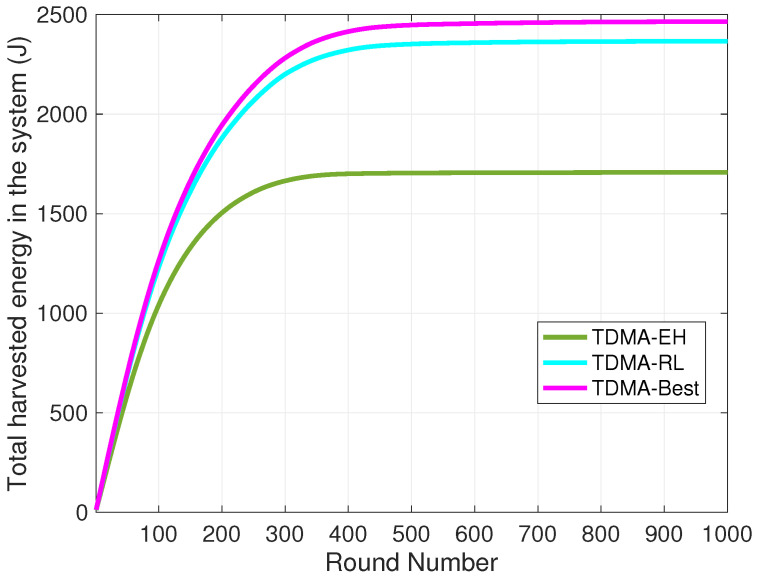
Total harvested energy in system in rounds.

We present the comparison of total harvested energy in the system in Figure 7. The TDMA-Best curve represents the maximum total energy captured by all nodes in the system, assuming nodes know the least efficient transmission slots to harvest energy in advance. This curve serves as an upper bound for the total energy available in the environment. Since energy harvesting is not incorporated in the TDMA-none method, we compare the TDMA-EH and TDMA-RL methods with this upper bound of available energy. As depicted in the plot, TDMA-RL captures 96% of the available energy in the system, while the TDMA-EH curve reveals that the total amount of energy in the system can only utilize 56% of the available energy.

**Figure 8:**
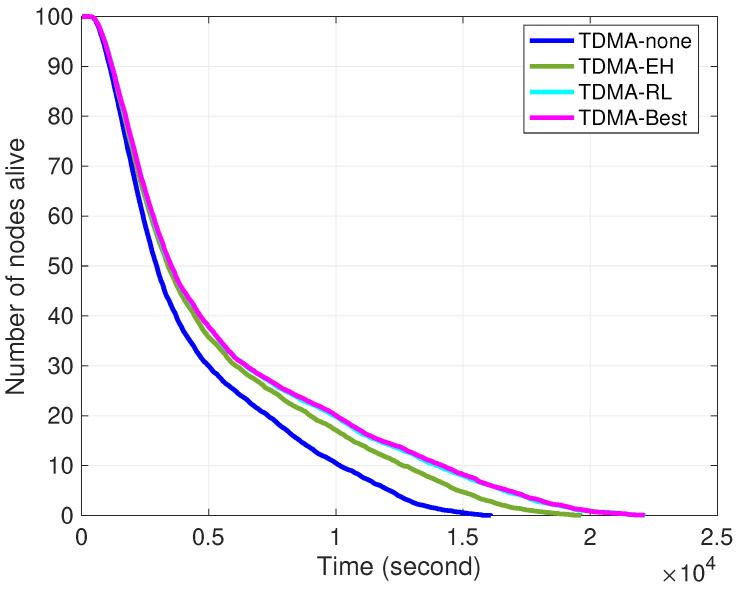
Number of alive nodes over simulation time.

**Figure 9:**
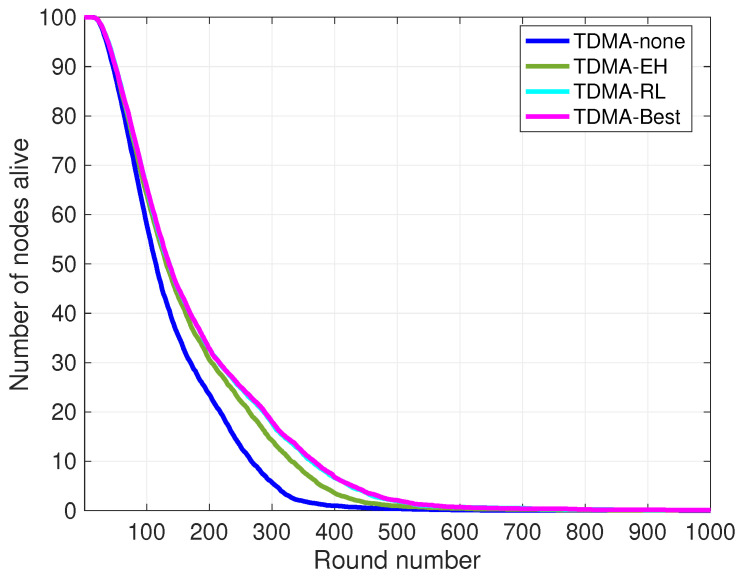
Number of alive nodes over simulation rounds.

Figure 8 and Figure 9 depict the performance of cooperative-learning-based slot selection to the traditional TDMA protocol on a lifetime basis. Considering the rounds consist of multiple frames, the duration of each round varies depending on the number of alive nodes. Both figures acknowledge that lifetime is significantly improved in the case of cooperative selection of transmission slots in clusters. While energy harvesting improves the lifetime by adapting to the dynamic underwater environment and predicting the fluctuations in ambient energy, it is observed that transmission slots can be selected with the aim of maximizing the utilization of the available energy in the environment. Furthermore, individual transmission slot selection at each node minimizes energy waste from overhearing while also relaxing the need for strict synchronization. It is noted that in both figures, the curves for TDMA-Best and TDMA-RL exhibit marginal disparities, yet without significant alterations in the number of active nodes per unit time or across rounds. The network lifetime is defined as the duration from the initialization of the sensor network until the depletion of the last node’s battery. To represent the numerical result of 100 simulation runs, the performance of each method with statistics of 100 simulation runs is depicted in Table 2. The results presented in Table 2 clearly illustrate the beneficial effect of TDMA-RL in increasing the number of active nodes over the course of rounds and enhancing the overall longevity of the network.

Note that due to the amount of harvested energy and the early steps of learning at the beginning of the rounds, the TDMA-EH, TDMA-RL, and TDMA-Best methods have a similar central tendency in FND. HND is improved 14% due to the amount of harvested energy that incorporates the traditional TDMA method. HND in TDMA-EH is improved by 2% with the TDMA-RL method. As more energy is collected towards the last stages of rounds, we observe that the efficiency of our method on LND increased by 25% and 40% in TDMA-EH and TDMA-RL, respectively.

## 7. Discussion

Underwater sensor networks encounter unique challenges, such as long propagation delays, limited bandwidth, and high attenuation, which can significantly decrease the performance of communication protocols. Therefore, traditional contention-free methods, specifically time division multiple access (TDMA), are commonly used in underwater sensor networks due to their simplicity and robustness in such demanding environments. Furthermore, clustering methods often rely on TDMA due to their efficiency in scheduling intra-cluster communication, minimizing collisions and energy consumption, which is the primary concern for the power-constrained nodes in underwater conditions. On the other hand, the dynamic nature of underwater environments causes significant variability in energy harvesting conditions. In this study, our primary objective is to augment the accumulated energy of underwater sensor nodes by dynamically selecting TDMA time slots. We specifically emphasized the integration of harvesting awareness into the transmission slot selection strategy. Our investigation revolves around assessing the impact of harvesting-adaptive slot scheduling in response to the varying levels of available energy underwater. Our results show that both TDMA-EH and TDMA-RL improve the network’s initial robustness and longevity compared to the baseline TDMA method. The inclusion of energy harvesting in TDMA-EH extends the network’s operational period, reflected in higher minimum and mean values for first node dead (FND). TDMA-RL further enhances performance by dynamically adapting time slots based on available energy, thus better sustaining network operations over time. However, TDMA-RL includes the added complexity of reinforcement learning, we would expect it to outperform the simpler TDMA-EH consistently. This similarity might indicate that the learning mechanism is not significant in the earliest phases of node operation compared to what is achieved by simple energy harvesting alone. One future investigation is a guided exploration strategy to quickly identify good actions.

In terms of half node dead (HND), TDMA-EH and TDMA-RL significantly outperform the baseline, indicating that they can maintain a larger portion of the network for longer periods. TDMA-RL, in particular, shows a slightly higher mean HND, demonstrating the advantages of adaptive scheduling. On the other hand, TDMA-RL shows a higher standard deviation in both HND (24.324) and LND (198.45) compared to TDMA-EH (21.968 and 168, respectively). This suggests greater variability in network performance, which might be due to the adaptive nature of the method leading to inconsistent outcomes across different simulation runs. While adaptive methods can optimize performance, it also shows that they can introduce variability. Q-learning, especially in the initial stages where the algorithm is still learning and exploring the environment. The last node dead (LND) results further confirm the superiority of these advanced methods, with TDMA-RL achieving the highest median and mean LND values, indicating its effectiveness in prolonging the overall network lifetime. The TDMA-Best method serves as an ideal benchmark, setting the highest performance targets and illustrating the maximum potential of the network with optimal time-slot assignments. This benchmark underscores the effectiveness of the TDMA-RL method, showing that adaptive learning strategies can approach optimal performance levels.

Overall, the study highlights the significant benefits of incorporating energy harvesting and adaptive scheduling into UASNs. TDMA-RL stands out for its ability to dynamically manage energy and time-slot allocation, leading to substantial improvements in network longevity and efficiency. The results suggest promising directions for future research, focusing on refining these methods and integrating advanced communication technologies to further enhance network performance and reliability.

## 8. Conclusions

The main issues that influence the design of underwater communication protocols are the battery life of underwater sensors and the dependability of low-rate acoustic communications. In this work, we used a stochastic model for piezoelectric energy harvesting and took into account the dynamic nature of underwater ambient resources to optimize the transmission schedule of the nodes. Our method considers spatio-temporal uncertainty in the available energy of the piezoelectric energy harvesting method. Our simulation results demonstrate that the proposed adaptive scheme, based on the decision of time slots to perform transmission, extends the network’s lifespan. An approach, distributed cooperative multi-agent reinforcement learning is proposed, considering the realistic channel and environmental specifications. Nodes interact with the environment to learn energy harvesting opportunities and select their transmission times with an adaptive strategy. Our main focus is exploiting the available ambient energy while alleviating the load on the cluster head. Performance evaluations reveal that our proposed approach improves the lifetime of the network by 15%, while the incorporation of energy harvesting only contributes 10% to the lifetime. Our study aims to provide valuable insights for researchers investigating optimal time slot allocation for the sustainable operation of UASNs. Towards this aim, we show the contribution of energy harvesting to the traditional contention-free medium access methods.

## 9. Future Work

As part of our future work, the overhead of efficient information sharing and decentralized coordination mechanisms will be investigated while maintaining effective coordination among sensor nodes. A comparison of our harvesting-aware method in terms of throughput and energy utilization, thereby providing a comprehensive evaluation of our proposed approach, is planned for future work. Moreover, the efficiency and performance of our transmission slot selection strategy can be improved with advanced multi-agent reinforcement learning algorithms, such as proximal policy optimization (PPO) and deep Q-networks (DQN), and their complexity, will be investigated with a varied size of the network. Additionally, we intend to incorporate variations in channel state to offer a more realistic approach that accounts for the influence of energy harvesting. On the other hand, we plan to validate our proposed method in this study regarding accuracy and reliability to model real-world underwater sensor network scenarios. We plan to compare our simulation results with experimental data obtained from field deployments or test-bed environments, particularly under various environmental conditions and operational scenarios.

Underwater communication encounters significant delays, higher bit error rates, and limited bandwidth. As a result, it requires adaptive and robust reinforcement learning strategies for time slot allocation. In contrast, terrestrial wireless sensor networks benefit from higher speeds, lower BER, and greater bandwidth, enabling more aggressive and efficient slot allocation through learning algorithms. In another area of future research, we will conduct a comprehensive analysis of delay performance in the adaptive time slot allocation approach, particularly in dynamic underwater environments. This will involve detailed modeling and simulation of delays associated with different slot allocation strategies, as well as empirical validation through field experiments. Our goal is to understand the trade-offs between delay, energy harvesting efficiency, and network throughput to enhance the overall performance of underwater acoustic sensor networks.

Moreover, the rapid development of new communication techniques such as rate-splitting multiple access (RSMA) and non-orthogonal multiple access (NOMA) presents opportunities. Furthermore, hybrid approaches, combining the strengths of multiple methodologies, such as integrating TDMA with non-orthogonal multiple access (NOMA) techniques, are also promising. To date, these techniques have not been extensively investigated in underwater communication systems. As a future research direction, their potential applicability in underwater environments may be explored. By pursuing these research directions, we aim to contribute to the advancement of UASNs, enhancing their performance and efficiency through innovative communication techniques and more accurate modeling of environmental factors.

## Figures and Tables

**Table 1:** Simulation Parameters.

System & Network Parameters	Definition	Value
$N_{node}$	Number of nodes	100
*M*	Network Size (m^3^)	250×250×250
*h*	Network Depth (m)	250
$S_{\left(x,y,z\right)}$	Sink Location	(125, 125, 0)
$T_{round}$	Round Duration (s)	100
**Channel & Modem Parameters **		
R	Data rate (kbps)	2
$E_{0}$	Initial Energy (kJ)	0.1
$P_{rx}$	Reception Power (W)	1.3
$P_{idle}$	Idle Power (mW)	2.5
*f*	Frequency (kHz)	25
$p_{ij}^{b}$	Bit error rate on link-(i,j)	$10^{-10}$
L	Packet size in frames (bits)	1024
κ	Spreading Factor	1.5
$B_{N}$	Noise Bandwidth (kHz)	1
$E_{DA}$	Data Aggregation Energy (nJ/bit/packet)	5
**Stochastic Energy Harvesting** **Model Parameters**		
*L*	Cantilever length (mm)	50
*B*	Cantilever width (mm)	3
$\varepsilon_{r}$	Relative permittivity	4000
$\varepsilon_{0}$	Absolute permittivity (F/m)	$8.85\times 10^{-12}$
$T_{pzt}$	Thickness (μm)	60
$d_{31}^{p}$	Piezoelectric constant (C/N)	$300\times 10^{-12}$
$S_{t}$	Strouhal number	0.2
$v_{f}$	Mean Flow Velocity (m/s)	0.65
ρ	Density of Ocean Water(kg/m^3^)	1000
λ	Mean event rate	5

**Table 2:** Network Lifetime over 100 run of simulations.

FND	Min	Max	Median	Mean	Std
TDMA-none	12	46	25	25	6.5758
TDMA-EH	13	48	25	26	7.1873
TDMA-RL	13	48	25	25	7.0877
TDMA-Best	13	46	26	26	7.0437
**HND**					
TDMA-none	81	154	113	114	17
TDMA-EH	90	192	128	131	21.968
TDMA-RL	91	202	128	134	24.324
TDMA-Best	90	201	132	135	24.39
**LND**					
TDMA-none	222	1340	355	388	138.36
TDMA-EH	249	1500	442	475	168
TDMA-RL	286	1500	498	536	198.45
TDMA-Best	285	1500	510	542	205.73

## Data Availability

Data are contained within the article.

## Abbreviations

The following abbreviations are used in this manuscript:

UASN	Underwater Acoustic Sensor Networks
EH-UASN	Energy Harvesting Underwater Acoustic Sensor Networks
TDMA	Time-division Multiple Access
CDMA	Code Divison Multiple Access
FDMA	Frequency Division Multiple Access
CSMA	Carrier-sense Multiple Access
RL	Reinforcement Learning
MAB	Multi-arm Bandit
LEACH	Low-Energy Adaptive Clustering Hierarchy
